# Rear-foot kinematics in runners with PFPS during walking, squatting and uphill running

**DOI:** 10.1186/1757-1146-5-S1-P26

**Published:** 2012-04-10

**Authors:** Jessica Leitch, Kathleen Reilly, Julie Stebbins, Amy B Zavatsky

**Affiliations:** 1 Department of Engineering Science, University of Oxford, Oxford, OX1 3PJ, UK; 2 Department of Physiotherapy, Nuffield Orthopaedic Centre, Oxford, OX3 7LD, UK; 3 Oxford Gait Laboratory, Nuffield Orthopaedic Centre, Oxford, OX3 7LD, UK

## Background

Patellofemoral pain syndrome (PFPS) is the most common overuse injury in distance runners. A pilot investigation found that runners with a history of PFPS exhibited increased rear-foot eversion and reduced rear-foot dorsiflexion compared to uninjured controls during level treadmill running [1]. The aim of the present study was to investigate whether these kinematic alterations were also demonstrated during activities that demanded more dorsiflexion (uphill running and squatting) and less dorsiflexion (walking) compared to level running.

## Materials and methods

Nine female runners with a previous history of PFPS and ten female controls participated in the study. Spherical reflective markers (9-mm) were attached to anatomical landmarks of both lower limbs [2]. A 12-camera Vicon MX System (Vicon Motion Systems, Oxford, UK) was used to collect 3-D spatial data at 200 Hz as the subject performed (i) five over-ground walking strides (self-selected speed), (ii) five squats and (iii) five uphill running strides on a treadmill (speed = 2.96 ms^-1^, incline = 10^o^). Rear-foot joint angles were calculated using the Oxford Foot Model [2]. The five trials for each activity and each subject were normalised to the stance (squat) period using cubic spline interpolation. Discrete kinematic parameters (peak rear-foot dorsiflexion, dorsiflexion excursion, peak rear-foot eversion) were identified for each of the five trials of each subject. The variables were compared between groups using one-tailed *t*-tests with an alpha level set at 0.05.

## Results

Subjects with a history of PFPS demonstrated significantly less dorsiflexion (peak) during walking and squatting compared to uninjured controls (Table 1). Dorsiflexion excursion was significantly lower and rear-foot eversion significantly higher in subjects with a history of PFPS compared to uninjured controls during uphill running.

**Table 1:** Kinematic parameters during walking, running and squatting for subjects with a history of PFPS (P) and uninjured controls (N). * Indicates a statistically significant difference (*p* < 0.05).

	Walk			Uphill Run			Squat		
Angle (^o^)	N	P	*p-*value	N	P	*p-*value	N	P	*p-*value

Peak dorsiflexion	11.2 (3.2)	7.9 (2.8)	0.01*	18.9 (7.2)	15.9 (2.5)	0.13	25.0 (8.2)	19.0 (3.3)	0.03*
Dorsiflexion excursion	17.0 (2.5)	15.1 (2.8)	0.06	17.5 (3.8)	14.0 (2.3)	0.04*	19.5 (8.8)	17.0 (2.5)	0.21
Peak eversion	5.8 (6.2)	9.5 (3.3)	0.07	7.1 (7.5)	12.8 (3.1)	0.03*	6.5 (10.8)	10.1 (3.2)	0.18

## Conclusion

The kinematic alterations that had been observed in subjects with a history of PFPS during level running [1] were also apparent during walking, uphill running and squatting. Further investigations to understand the relationship between rear-foot joint motion and patellofemoral joint kinematics are required.
